# Use of left atrial appendage as an autologous tissue source for epicardial micrograft transplantation during LVAD implantation

**DOI:** 10.3389/fcvm.2023.1143886

**Published:** 2023-04-28

**Authors:** Jan D. Schmitto, Aleksi Kuuva, Kai Kronström, Jasmin S. Hanke, Esko Kankuri

**Affiliations:** ^1^ Department of Cardiac-, Thoracic-, Transplantation and Vascular Surgery, Hannover Medical School, Hannover, Germany; ^2^ EpiHeart Oy, Helsinki, Finland; ^3^ Department of Neuroscience and Biomedical Engineering, Aalto University, Espoo, Finland; ^4^ Department of Pharmacology, Faculty of Medicine, University of Helsinki, Helsinki, Finland

**Keywords:** autologous cardiac micrografts, cell therapy, epicardial transplantation, left ventricular assist device (LVAD), left atrial appendage (LAA), heart failure

## Abstract

We report here the first clinical use of the left atrial appendage (LAA) for epicardial micrograft transplantation during left ventricular assist device (LVAD) implantation. Previously, a sample from the right atrial appendage (RAA) has been available for processing and administering micrograft therapy in cardiac surgery. Both LAA and RAA are rich sources of various types of myocardial cells and are capable of providing both paracrine and cellular support to the failing myocardium. The surgical approach of LAA micrografting facilitates epicardial micrograft therapy dose escalation and treatment of larger myocardial areas than done previously. Moreover, as collection of treated vs. untreated tissues from the recipient heart is possible following LVAD implantation at the time of heart transplantation, the evaluation of the therapy's mechanism of action can be further deciphered at cellular and molecular levels. This LAA modification of the epicardial micrografting technique has the overall potential to facilitate the adoption of cardiac cell therapy during heart surgery.

## Introduction

Myocardial recovery and device explantation are achieved in 3% of patients receiving mechanical circulatory support by left ventricular assist device (LVAD). It is considered that LVAD-induced cardiac unloading induces reverse cardiac remodeling, which contributes to myocardial recovery (1–4). Further possibilities for inducing structural and functional improvements in the myocardium are offered by cellular therapies, which have been under evaluation for the treatment of heart failure over two decades (5, 6). Despite controversies, cellular therapies remain under investigation for their numerous potential beneficial effects on the heart (7, 8). Theoretically, inducing myocardial regeneration at the cellular level can improve myocardial recovery after LVAD implantation. The high costs of cell therapy manufacturing, extensive culturing, dedicated facilities required, and concerns of mutations due to prolonged extensive expansion and *in vitro* manipulations have attracted the search for alternative possibilities to cell therapy production and administration. During LVAD implantation surgery, autologous cardiac tissue can be harvested for the purpose of cell therapy generation from, for example, atrial appendages and left ventricular tissue. Recent data have shown that epicardial transplantation of autologous right atrial appendage (RAA) micrografts is clinically safe and feasible and is associated with beneficial structural and functional effects on ischemic myocardium (9–11).

As compared to the limited amount of tissue that can be harvested from the RAA for therapy preparation, the left atrial appendage (12, 13) allows for harvesting of a substantially greater amount of tissue and thus serves as a scalable tissue source for autologous therapeutic applications. We provide here the first clinical insight into the feasibility and safety of autologous left atrial appendage (LAA) harvesting, processing, and epicardial micrograft transplantation during LVAD implantation.

## Method

A 61-year-old male patient with dilated cardiomyopathy underwent LVAD implantation as destination therapy. Prior to surgery, the patient was provided with information regarding the implantation procedure. The patient provided written informed consent for the procedure and follow-up. For the procedure, a HeartMate 3 LVAD (Abbott, Green Oaks, IL, United States) was implanted *via* full sternotomy. The LAA was then closed (Atriclip, Atricure Inc., Mason, OH, United States), removed, and forwarded for mechanical disaggregation and epicardial graft composition. A set of instrumentation for micrografting including a tissue processing station (EpiHeart Oy, Helsinki, Finland) and micrografting tool (HBW srl, Turin, Italy) were used.

**Figure 1:**
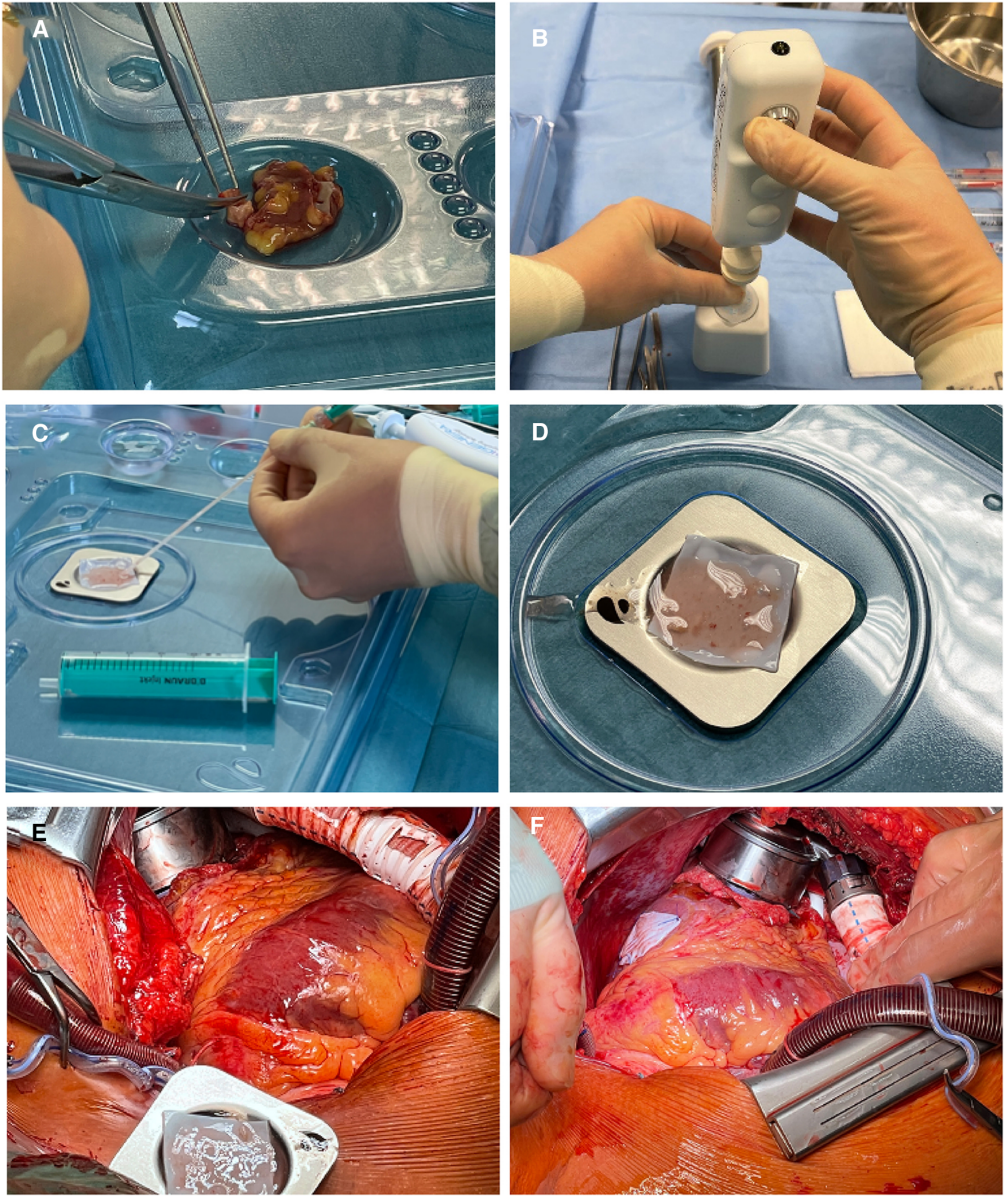
Left atrial appendage processing and epicardial transplantation. (**A**) Pieces of LAA are cut for processing. (**B**) Mechanical processing of LAA pieces to micrografts. (**C**) Application of LAA micrografts to the ECM sheet. (**D**) ECM sheet with LAA micrografts waiting for transplantation. (**E**) Transplant in transplant holder for flipping onto the epicardial surface. (**F**) Epicardially applied LAA micrograft patch in place on the left ventricle. LAA, left atrial appendage; ECM, extracellular matrix.

Figure 1 illustrates LAA processing and epicardial LAA micrograft patch application. The harvested LAA tissue (∼5 g) was first cut into smaller pieces. Processing into micrografts was carried out from 1 g of LAA tissue with the micrografting tool in four 1-min cycles. Each cycle was followed by micrograft harvesting in 4 ml of cardioplegia into a 50-ml centrifugation tube. To ensure washing in an ample buffer solution volume, fresh cardioplegia was added to the tube to reach a total volume of 25 ml. After centrifugation and removal of the cardioplegia supernatant, the LAA micrograft pellet was suspended in saline-diluted (1:1) fibrinogen component of the Tisseel fibrin sealant (Tisseel, Baxter Healthcare Corp., Westlake Village, CA, United States). The micrografts were then applied onto a decellularized equine pericardium matrix sheet (Auto Tissue Berlin GmbH, Berlin, Germany) spread on a dedicated transplant holder (EpiHeart Oy). The completed transplant was placed on a cooling plate (EpiHeart Oy) on the transplant holder to minimize micrograft metabolic activity before transplantation and to allow for controlled fibrin mesh formation. Saline-diluted (1:30) thrombin component of the fibrin sealant was then used to induce the formation of a fibrin mesh gel to support and fix the LAA micrografts to the matrix sheet. At the end of surgery, the autologous LAA micrograft transplant was fixed onto the epicardial surface with four sutures.

## Results

The harvesting, mechanical tissue disaggregation, and preparation of the epicardial graft was carried out in a total of 35 min. Impact of the process on the duration of surgery was an increase of 6 min: 2 min for collecting the atrial appendage and 4 min for fixing and suturing the graft in place on the epicardial surface. The postoperative course was uneventful, and the patient was discharged home on postoperative day 33 with regular outpatient follow-up after approximately 3 months.

## Discussion

We report here the first clinical application of intraoperative cardiac tissue micrografting using the LAA. The LAA micrograft transplant was successfully applied epicardially in conjunction with LVAD implantation. In addition to potential therapeutic benefits, this approach facilitates obtaining mechanistic proof of remodeling efficacy at functional, molecular, and structural levels using clinical imaging tools. Importantly, this protocol enables in-depth analytical comparisons of a baseline myocardial sample (available from the apical myocardium removed for LVAD implantation) with samples from treated and untreated myocardial areas available from the discarded host heart at heart transplantation. Such analyses can provide unique insights into intriguing new paracrine and cellular mechanisms of action of epicardial cellular therapies and increase our understanding on the nonthrombogenic roles of the LAA (14). Interestingly, graft-to-myocardium migration of tissue-resident macrophages was associated with myocardial healing after epicardial transplantation of LAA in a mouse model of acute myocardial infarction and heart failure (15). The contribution of graft-to-myocardium cell migration to myocardial healing will be an exciting topic for further research the autologous atrial appendage micrografts.

Similar therapy utilizing a small piece of the RAA has already been evaluated in conjunction with coronary artery bypass grafting (CABG) surgeries (9, 11). With increased amounts of tissue, for example from the LAA, available for processing, larger areas can be treated, and increased doses can be administered. Moreover, additional tissues utilizable for cell therapy are also available from cardiac surgeries. In addition to atrial appendage tissues, even leftover parts of vascular grafts at CABG surgery and apical myocardial tissue removed for the LVAD inlet at LVAD implantation are available for rapid mechanical processing to micrografts and subsequent epicardial transplantation intraoperatively. Such hybrid-tissue micrograft transplants may offer unprecedented therapeutic advances.

The adaptation of epicardial micrografting and the utilization of LAA, as presented here, offer a relatively ample autologous cardiac tissue source for surgical cell therapy. Straightforward mechanical disaggregation and available regulatory-approved devices are expected to facilitate the adoption of this cardiac cell therapy approach. This, in turn, enables further therapy optimization and provides possibilities to obtain advanced mechanistic understanding of therapy efficacy.

## Data Availability

The original contributions presented in the study are included in the article, further inquiries can be directed to the corresponding author.
